# Special Section Guest Editorial: Short Wave Infrared Techniques and Applications in Biomedical Optics

**DOI:** 10.1117/1.JBO.28.9.094800

**Published:** 2023-09-09

**Authors:** Daniel Fried, Mark Pierce, Darren Roblyer

**Affiliations:** a University of California San Francisco, School of Dentistry, San Francico, California, United States; b Rutgers University, Department of Biomedical Engineering, Piscataway, New Jersey, United States; c Boston University, College of Engineering, Boston, Massachusetts, United States

## Abstract

The editorial introduces the JBO Special Section on Short Wave Infrared Techniques and Applications in Biomedical Optics.

We are pleased to introduce a special section of JBO, on Short Wave Infrared Techniques and Applications in Biomedical Optics. The short wave infrared (SWIR) spectral region (approximately 1000-2000 nm) offers several unique characteristics that may be beneficial in biomedical applications. At wavelengths beyond the visible and near-infrared (NIR) regions, scattering in tissue is lower, autofluorescence is reduced, and water, lipids, and collagen all exhibit distinct spectral absorption features.

To-date, the development of biophotonic techniques and applications at SWIR wavelengths has been relatively unexplored. Several factors including cost, performance, and availability of SWIR light sources and detectors, a lack of SWIR-emitting contrast agents, and limited tools for simulations and modelling have likely contributed to this situation. In recent years, advances in these and other areas have opened the door to new biophotonics research using SWIR wavelengths. This special section features an exciting collection of papers on topics ranging from new optics and instrument development to pre-clinical and clinical applications using SWIR light.

Imaging at SWIR wavelengths can require the design and fabrication of new optical elements to optimize performance and minimize aberrations. Fabricating small diameter refractive elements with a large field-of-view for endomicroscopy is additionally challenging. Xie et al. designed and fabricated a transmissive metalens for single fiber intravascular imaging at 1310 nm and demonstrated superior imaging performance over refractive optics (https://doi.org/10.1117/1.JBO.28.9.094802).

Fluorescence lifetime imaging (FLIM) provides additional contrast that is complementary to traditional fluorescence intensity imaging. Chavez et al. demonstrated FLIM at SWIR wavelengths (>1000 nm) by using the SWIR emission tail of two commercial NIR probes and two commercial SWIR probes (https://doi.org/10.1117/1.JBO.28.9.094806). Differences in the fluorescence lifetimes of these four probes allowed multiplexed SWIR FLIM to be achieved on a time-domain mesoscopic fluorescence molecular tomography platform.

In preclinical studies, background autofluorescence can significantly hamper the ability to visualize small tumors in *in vivo* models. Sun et al. examined the effects of diet, excitation, and emission wavelengths on background autofluorescence at NIR and SWIR wavelengths (https://doi.org/10.1117/1.JBO.28.9.094805). The authors showed that imaging using SWIR emission wavelengths (1000-1600 nm) significantly reduced autofluorescence and increased signal-to-background when imaging with indocyanine green, without the need to modify the animal’s diet.

The low scattering and low autofluorescence at SWIR wavelengths can allow for high contrast, deep tissue imaging. Su et al. demonstrate a platform for rotational single camera stereo imaging of small animals labeled with a SWIR emitting fluorescent polymer dot contrast agent (https://doi.org/10.1117/1.JBO.28.9.094807). The use of wavelengths longer than 1300 nm enabled 3D reconstruction of subsurface tumor vasculature with 150  μm spatial resolution at 5 mm depth.

Multiple papers in this special section illustrate the potential for translation of SWIR techniques into clinical applications. Klein et al. designed and built a rigid endoscope for imaging within the human nasal cavity to detect cerebrospinal fluid (CSF) leakage (https://doi.org/10.1117/1.JBO.28.9.094803). The authors identified the major SWIR absorption bands of CSF and used narrowband reflectance imaging at 1480 nm to identify CSF in *ex vivo* studies.

Fluorescence guided surgery has demonstrated its potential for assisting surgeons in identifying tumor margins and tissue structures at visible and near-infrared wavelengths. Waterhouse et al. (issue cover) developed a multispectral SWIR fluorescence imager for fluorescence guided surgery (https://doi.org/10.1117/1.JBO.28.9.094804). In a preclinical model of neuroblastoma, the authors labeled tumor cells with Dinutuximab-IRDye800 and imaged the tail of the emission spectrum at six wavelength bands between 850-1350 nm. Machine learning algorithms classified tissue as tumor, non-tumor, or background with 97.5% per-pixel accuracy.

SWIR imaging is appealing for dental applications because these wavelengths are not affected by tooth staining. Chang et al. used SWIR reflectance imaging at 1950 nm in combination with optical coherence tomography and thermal imaging to assess enamel structure and composite restorations (https://doi.org/10.1117/1.JBO.28.9.094801). The strong optical absorption of water allowed differences in diffusion rates during dehydration to be identified in sound and demineralized enamel, potentially enabling clinical monitoring of secondary caries lesions.

Finally, SWIR wavelengths also offer new possibilities for continuous physiological monitoring. Spink et al. designed a wearable probe using light-emitting diode illumination at 980 nm, 1200 nm, and 1300 nm with four source – detector separations to measure the diffuse reflectance of skin (https://doi.org/10.1117/1.JBO.28.9.094808). In a phantom study, a deep neural network determined water and lipid content with errors below 1.5%. This prototype wearable device will enable future human subject studies with continuous monitoring for dialysis patients, treatment response monitoring in cancer, and athletic performance tracking.

In summary, SWIR techniques and applications are rapidly growing from basic science studies through to clinical applications. We expect this field will continue to grow as new enabling components reach the market, and as new instruments and systems align with unmet clinical needs. We express our appreciation to the authors who contributed their research to this special section, and to the reviewers for their valuable feedback on each manuscript. We also greatly appreciate the work of Senior Editor Renae Keep for her guidance throughout the curation of this special section.

